# Association studies using family pools of outcrossing crops based on allele-frequency estimates from DNA sequencing

**DOI:** 10.1007/s00122-014-2300-4

**Published:** 2014-03-26

**Authors:** Bilal H. Ashraf, Just Jensen, Torben Asp, Luc L. Janss

**Affiliations:** 1 Department of Molecular Biology and Genetics, Centre for Quantitative Genetics and Genomics, Aarhus University, Blichers Alle 20, Post Box 50, 8830 Tjele, Denmark; 2 Department of Molecular Biology and Genetics, Aarhus University, Forsogsvej 1, 4200 Slagelse, Denmark

## Abstract

*****Key message***:**

**We propose a method in which GBS data can be conveniently analyzed without calling genotypes.**

**Abstract:**

F2 families are frequently used in breeding of outcrossing species, for instance to obtain trait measurements on plots. We propose to perform association studies by obtaining a matching “family genotype” from sequencing a pooled sample of the family, and to directly use allele frequencies computed from sequence read-counts for mapping. We show that, under additivity assumptions, there is a linear relationship between the family phenotype and family allele frequency, and that a regression of family phenotype on family allele frequency will estimate twice the allele substitution effect at a locus. However, medium-to-low sequencing depth causes underestimation of the true allele substitution effect. An expression for this underestimation is derived for the case that parents are diploid, such that F2 families have up to four dosages of every allele. Using simulation studies, estimation of the allele effect from F2-family pools was verified and it was shown that the underestimation of the allele effect is correctly described. The optimal design for an association study when sequencing budget would be fixed is obtained using large sample size and lower sequence depth, and using higher SNP density (resulting in higher LD with causative mutations) and lower sequencing depth. Therefore, association studies using genotyping by sequencing are optimal and use low sequencing depth per sample. The developed framework for association studies using allele frequencies from sequencing can be modified for other types of family pools and is also directly applicable for association studies in polyploids.

**Electronic supplementary material:**

The online version of this article (doi:10.1007/s00122-014-2300-4) contains supplementary material, which is available to authorized users.

## Introduction

Gene mapping, by linkage or association studies, is well established in diploid organisms, with individual measurements of phenotypes, and in inbred lines such as RIL populations (Lander and Botstein 1989; Ripol et al. 1999; Lon and John 2001; Andersen and Lübberstedt 2003). This still leaves areas where gene mapping is less straightforward, for instance in cases where phenotypes are measured on groups of individuals, such as yields of plots, and where the species is cross-pollinating. This is for instance the situation in breeding of perennial ryegrass (*Lolium Perenne* L.). This species is extensively used as forage and turf grass in Europe and is the most valuable forage and turf grass species in temperate climates (Altpeter et al. 2000). Although some phenotypes in perennial ryegrass can be measured on individual plants, traits such as yield and persistency are frequently measured under competitive sward conditions.

In an outcrossing species it is not straightforward to link the yield obtained on a family to a genotype, because the individuals within a family are genetically heterogeneous (Huff 1997; Thorogood et al. 2002). Thus, either the family yield must be linked to the genotypes of the parents or some kind of compound/average “family genotype” must be obtained. We argue here that for diploid outbreeding plants the full-sib family will show up to four dosages of every allele, the sum of up to two-allele dosages present in each parent. Thus, the “family genotype” of an F2 full-sib family can be described as a tetraploid genotype. This allows developing a framework for association studies using F2 family phenotypes and genotypes.

For the genotyping of family pools, a sequencing approach is considered here. Next-generation sequencing, with its increasing throughput and rapidly decreasing costs, has become a feasible approach for genotyping (Deschamps et al. 2012). Two sequencing-based approaches for genotyping have been proposed so far: (1) complexity reduction by sequencing of a limited part of the genome from restriction sites, called genotyping-by-sequencing (GBS) (Elshire et al. 2011); and (2) whole-genome sequencing (WGS) (John et al. 2011). GBS can be considered when no reference genome is available, but would need a reasonable sequencing depth, probably 5–10× as a minimum average depth, to avoid too many missing data points. WGS with very low sequencing depth (<1×) has been proposed as a genotyping strategy in humans, and thus relies on an available full-genome reference sequence and individual full-genome sequences to impute large amounts of missing data (Pasaniuc et al. 2012). Such resources, however, are not yet available for perennial ryegrass at this moment, leaving GBS as the currently viable approach for high-throughput genotyping. The work in this study is based on this background, where we consider average sequencing depths of 5× and higher.

GBS technology will be interesting for measurements on pools because it primarily obtains allele counts from the sequencing reads, which can be processed to allele-frequency estimates (Byrne et al. 2013). Ideally, when the data are from pools, such allele-frequency estimates would be used directly, rather than calling genotypes. Accurate calling of tetraploid genotypes from sequence data requires a sequencing depth of 60×–80× (Uitdewilligen et al. 2013), which would make GBS too expensive for large-scale genotyping in the application we consider. Arguably, the allele frequencies also suffer from inaccuracy, but we show here that we can take account of that inaccuracy when working with allele frequencies by correcting for the measurement error. Measurement error on covariates is well known to cause underestimation of the regression coefficient (Bekker 1986; Chesher 1991), but correcting for this measurement error when using GBS data is not yet described. The use of allele-frequency estimates from pools for association and linkage studies has been considered before, but only in the context of pooling based on phenotypes, e.g. high/low phenotype pools (Sham et al. 2002; Zou and Zhao 2005) or cases/control pools (Norton et al. 2004; Moskvina et al. 2005). The case where pools are families, and where one phenotype per family is measured, is not yet considered, and theory and models for association studies based on allele frequencies are lacking for this case.

The aim of this study is to develop an approach for association studies using F2-family pools based on allele-frequency estimates from GBS data, and to study the optimal design for an association study using GBS. The ultimate goal of this work is to supply methods for association studies in breeding material of outbreeding species that use family-based breeding systems, such as several grass species. For the optimal design, we consider the usual practical constraint where the total sequencing budget is fixed. This implies that a balance needs to be found in the number of samples, sequencing depth and number of SNPs, where increasing one will go at the expense of others. We will use one- and two-locus simulation studies to verify allele effect estimates and to determine optimal design. We also develop a correction for the measurement error from GBS data that leads to underestimation of the allele effect. We will develop our framework by considering the estimation of the additive allele effect and using simulations for a continuous normally distributed trait. The linear model framework allows to extend this straightforwardly to include environmental effects, multiple SNPs and interactions of various kinds, and to consider other distributions for the trait.

## Materials and methods

### Notation for individuals

First, we develop notation for a one-locus model in diploid individuals, which will apply to parents in a perennial ryegrass breeding program. For a biallelic locus, genotypes are denoted as *aa*, *Aa*, and *AA*, with matching numerical values as the allele dose for the *A* allele as *g* = {0, 1, 2}. The genotypic value *G* is expressed as *0*, *a* and 2*a*. Further, the frequency of the *A* allele is taken as *p* in parents, and assuming Hardy–Weinberg equilibrium which implies assumption of random mating, the genotype frequencies are (*1* − *p*)^2^, 2*p*(*1* − *p*) and *p* ^2^. This leads to the well-known expression E[*g*] = 2*pa* and genetic variance for individuals at the biallelic locus, assuming that parents are unrelated and non-inbred (e.g., Falconer and Mackay 1996):

1 $${\text{Var}}\left[ G \right] \, = \, 2p\left( {1 - p} \right) \, a^{2}$$

### Genotype of F2-family pools

**Fig. 1:**
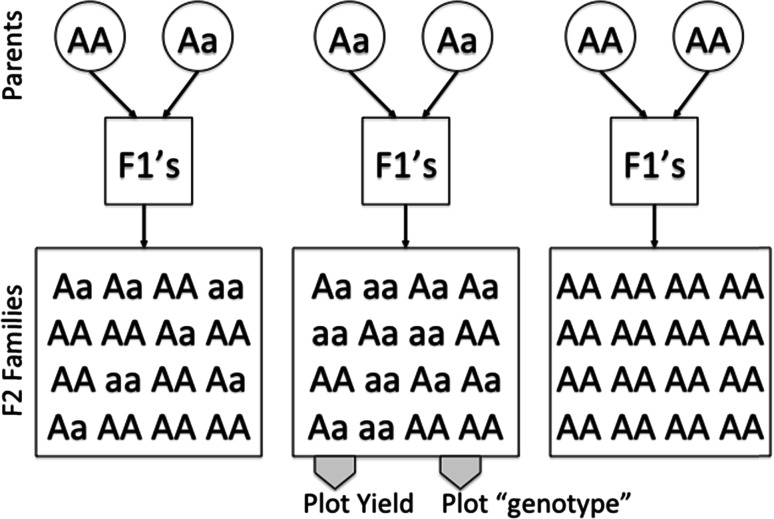
Schematic representation for the creation of family pools used to measure phenotypes such as yield in grasses: three crosses are shown with parents that segregate at a biallelic locus with alleles *a* and *A*; the created F2 families will segregate in five distinct segregation ratios with allele frequencies within the families of 0, ¼, ½, ¾, and 1, which corresponds to the combined allele dosage in the two parents of each family

Next, we determine an “average family genotype” with the ultimate aim to associate family phenotypes with such average family genotypes. Figure 1 shows a schematic representation of the creation of F2 families as used in ryegrass breeding. Three parental matings producing three F2 families are shown, with segregating genotypes in parents and F2 families. In this scheme, due to the intermediate F1 generation, genotypes in the F2 families segregate in Hardy–Weinberg proportions, assuming random cross-pollination between the F1’s. The segregation ratios in the F2 families seem to match the average allele dosage in the parents. For instance, the left shown mating with a total allele dosage of 3 *A* alleles (out of 4) in parents shows genotype segregation in the F2 family at a ¾ allele frequency for the *A* allele (1/16 *aa*, 6/16 *Aa*, 9/16 *AA*). We propose here to define an F2-family genotype as either the average allele dosage in the pool, which will be from 0 to 2 in steps of ½, or as the allele frequency within that family, in quarters. Table 1 lists all possible parental matings, F1 and F2 genotypes, and the defined F2-family genotype as an allele frequency. The F2-family genotype can, conceptually, also be thought of as a tetraploid genotype.

**Table 1:** F2 pool allele frequencies obtained from parental crosses

Parental mating	Frequency of mating	F1 genotypes	Average pool allele dose	F2 genotypes	F2 pool alelle frequency (*g*_F2_)
aa × aa	(1 − p)^4^	aa	0	aa	0
aa × Aa	4*p*(1 − *p*)^3^	aa (1/2) and Aa (1/2)	1/2	aa (9/16), Aa (6/16), AA (1/16)	1/4
aa × AA	2*p* ^2^(1 − *p*)^2^	Aa	1	aa (1/4), Aa (1/2) and AA (1/4)	1/2
Aa × Aa	4*p* ^2^(1 − *p*)^2^	aa (1/4), Aa (1/2) and AA (1/4)	1	aa (1/4), Aa (1/2) and AA (1/4)	1/2
Aa × AA	4p^3^(1 − *p*)	Aa (1/2) and AA (1/2)	3/2	aa (1/16), Aa (6/16), AA (9/16)	3/4
AA × AA	*p* ^4^	AA	2	AA	1

Table 1 also lists the frequency at which every parental mating occurs. This mating frequency depends on the population allele frequency and assumes random sampling and random mating of parents with respect to the single locus considered. Because this mating frequency directly relates to the frequency for the five F2-family genotypes, these also can be thought of as the genotype frequencies for the F2-family genotypes. These are indeed genotype frequencies for a tetraploid genotype, which can be derived from a binomial expression to sample four alleles with frequency *p*:

2 $$P_{\text{r}} \left( k \right) = \frac{4!}{{k!\left( {4 - k} \right)!}}p^{k} (1 - p)^{4 - k} , \,\quad k = 0, \, \ldots \, 4$$

From expression (2), it can be directly obtained that the frequencies for the F2 pool genotypes {0, ¼, ½, ¾, 1} are (1 − *p*)^4^, 4*p*(*1* − *p*)^3^, 6*p* ^2^(1 − *p*)^2^ *, 4p* ^3^(*1* − *p*) and *p* ^4^.

### Association of family phenotypes and genotypes

From Table 1, it can also be verified that the additive genotypic value in the F2 pool is the average of the additive genotypic values of the parents, i.e. *G*_F2_ = ½ (G_P1_ + G_P2_). We assume that the family phenotype (the collective performance of the group) is the same as the average phenotype in a family (the average of the performance of the individuals). Thus, the genotypic value of an F2 pool can be expressed in the same way as for a biallelic genotype for an individual:

3 $$G_{\text{F2}} = g_{\text{F2}} a$$

The genetic variance across families explained by the F2 pool genotypes works out to be:

4 $${\text{Var}}\left( {G_{\text{F2}} } \right) = \left( {\text{var} \left( {\frac{1}{2}g_{\text{P1}} } \right) + \text{var} \left( {\frac{1}{2}g_{\text{P2}} } \right)} \right) = p(1 - p)a^{2}$$

This is half of the variance explained by genotypes measured on an individual. This expression for the variance assumes no covariance between the parental genotypes, i.e. assumes that parents are not related, but the expression could be extended to include such a covariance to account for relationship between the parents.

### Estimate of allele effect in F2-family pools

In general, the allele effect at a locus is estimated by regressing phenotypes on the genotype covariate. The expectation of this regression can be derived by expressing the phenotype as the sum of genotypic value and an environmental term:

5 $$P = G + E$$

For the case of F2 pools, the genotypic value included in the phenotype is $G_{\text{F2}} = \bar{g}_{\text{F2}} a$, which leads to a regression of F2 phenotypes on F2 pool genotypes from the model:

6 $$\begin{gathered} P = \mu + b_{\text{F2}} g_{\text{F2}} + e \hfill \\ {\text{where }} b_{\text{F2}} = \frac{{\text{cov} (P_{\text{F2}} ,g_{\text{F2}} )}}{{\text{var} (g_{\text{F2}} )}} = \frac{{\text{var} (g_{\text{F2}} )a}}{{\text{var} (g_{\text{F2}} )}} = 2a \hfill \\ \end{gathered}$$

This shows that the use of F2 pool frequencies will obtain twice the estimate of allele effect. However, the standard error on the estimate using F2 pools will be larger than when individuals could have been used, which is a consequence of having only half the variance across pools compared to individuals. Expression (6) assumes that the genotypes are obtained without error, which is generalized in the next section.

### Using genotyping by sequencing and genotypes with measurement error

When using GBS, genotypes will be subject to measurement error which leads to underestimation of the allele effect. For the use of GBS, we consider an approach where genotypes are not explicitly called, but allele frequencies obtained from sequencing are directly used for an association study. For a genotype obtained by GBS, consider that *S*_T_ total sequencing reads are obtained, with *S*_1_ reads showing one SNP allele and *S*_2_ reads showing the other SNP allele. This allows to directly obtain an estimate of the genotype in the form of an allele-frequency estimate, for instance arbitrarily for the first allele:

7 $$\hat{g}_{\text{F2}} = S_{1} /S_{\text{T}}$$

This genotype estimate will be subject to measurement error due to binomial sampling, which depends on population allele frequency. However, as we show below, the final expression for the bias in the allele effect estimate does not depend on population allele frequency. The average binomial sampling variance on these genotype estimates is determined by the underlying allele frequencies within the F2 families, which are the *g*_F2_ values in Table 1, weighted by the frequencies for these F2 families to occur, which is given by Eq. (2):

8 $$\sigma_{\text{bin}}^{2} = \mathop \sum \limits_{k = 1}^{5} g_{\text{F2}} \left( k \right)\left( {1 - g_{\text{F2}} \left( k \right)} \right)\Pr \left( k \right) = \frac{3p(1 - p)}{{4S_{\text{T}} }}$$

This allows to derive the expected estimate of allele effect from regressing F2 phenotypes on F2 pool allele frequencies, from the model:

9 $$\begin{gathered} P = \mu + b_{\text{F2}} \hat{g}_{\text{F2}} + e \hfill \\ b = \frac{{\text{cov} (P_{\text{F2}} ,\hat{g}_{\text{F2}} )}}{{\text{var} (\hat{g}_{\text{F2}} )}} = \frac{{\text{var} \left( {g_{\text{F2}} } \right)a}}{{\text{var} (g_{\text{F2}} ) + \sigma_{\text{bin}}^{2} }} \hfill \\ \end{gathered}$$

The crucial difference with expression (6) is that the denominator in (9) is increased by the binomial noise term. This shows that there is an underestimation or bias in the estimate of the allele effect of:

10 $${\text{bias}} = \frac{{\text{var} (g_{\text{F2}} )}}{{(\text{var} \left( {g_{\text{F2}} } \right) + \sigma_{\text{bin}}^{2} )}} = \frac{1}{{1 + \sigma_{\text{bin}}^{2} /\text{var} (g_{\text{F2}} )}} = \frac{1}{{1 + 3/S_{\text{T}} }}$$

where the last expression in (10) is based on using the binomial noise variance from (8) and the genotype variance in F2 pool genotypes, which is (4) omitting *a* ^2^. Equation (10) shows that the bias in the allele effect does no longer depend on allele frequency. The above expression (10) is derived for a constant sequencing depth across all samples. To account for variable depth across samples, the harmonic weighted mean of the depths per sample should be used in (10).

## Simulation setup

We performed simulation studies to verify estimation of allele effect and to study optimal design for an association study when the total sequencing budget is fixed. For estimation of the allele effect, we vary sample size, allele frequency and sequencing depth in a one-locus model and verify that underestimation of the allele effect as described by Eq. (10) only depends on sequencing depth. For the power study, we vary the sample size versus sequencing depth, and SNP density versus sequencing depth in such a way that the total sequencing effort is the same. Power studies are done in one-locus and two-locus models, where the two-locus model considers the case that we observed a marker linked to a causative mutation.

### One-locus model and estimation of allele effect

In the general case, the allele effect at a locus can be estimated by regressing phenotypes on the allele frequency. This regression can be described by expressing phenotype as the sum of genotypic values of F2 frequency pool and environmental term. In this simulation study, the allele effect was set as 1 and assumed that there is no other genetic variation except environmental standard deviation. The explained variance from the one-locus model (4) at three levels of given allele frequencies (0.1, 0.3 and 0.5) works out to be 0.0014, 0.0032, 0.0038 at environmental standard deviation 4. Normally, average sequencing depth is being used in most studies, but here we are keeping it as constant. R scripts used for these simulations are available in supplementary material. The following steps were made in this simulation:

1. Frequencies of F2 pool genotypes were generated using the binomial distribution expression (2), we used the sample sizes (number of families) 500, 1,000, 2,000 and 4,000 against the sequencing depth 3, 7, 15 and 30 and allele frequencies 0.1, 0.3 and 0.5. The environmental standard deviation in this model was set as 4 and 10 (Results at SD 10 can be seen in supplementary material Table 2). Here, the families are the observation units, because we have one observation per family. The generated counts were divided by full sequencing depth.
2. The sequencing counts were generated using true and observed frequencies across the families. The true frequencies give a best possible estimate if the frequency per family would be known without error and observed frequencies are the counts generated from the binomial distribution, so it is a reflection of that same frequencies, but with the noise from the binomial sampling.
3. To estimate the allele effect, we regressed F2 phenotypes on F2 pool genotypes using the model in expression (9) in the R lm-function (Chambers 1992).
4. The estimated regression coefficients were computed for true and observed frequencies (results with true frequencies presented in supplementary material Table 1).
5. The procedure was repeated 1,000 times; mean and standard deviation of the estimated regression coefficients were reported.

## One-locus model power study

We performed simulations to study the optimal design for association study in one-locus model. Here, we varied sample size, sequencing depth, allele frequency and environmental standard deviation. We repeated the same previous steps 1–2 and used regression of F2 phenotypes on F2 pool genotype expression (9) to obtain *P* values for both observed and true frequencies.

### Incorporating additional errors

To see how the power changes by including sequencing and genotype calling errors in simulation studies. Here, we simulate the situation in which power to detect a single gene associated with a marker in the presence of 10 % sequencing errors per read and genotype calling at 5 % level of significance. We used variable sequencing depth across the families, assuming a Poisson distribution with an average depth 3, 7, 15 and 30× for the family sizes 4,000, 2,000, 1,000 and 500, respectively. This whole procedure was replicated 1,000 times and the number of significant cases counted.

### Two-locus model power study

In a second simulation study, two loci in Linkage Disequilibrium (LD) were generated, where one locus was the causal but unobserved locus affecting the phenotype, and the second locus was an observed marker locus used for analysis. In this simulation, the level of LD between causal and marker locus was varied at three levels to show the impact of observing a linked locus instead of the causal locus. The SNP density, and thus average LD between SNPs and causal loci, can be modified in GBS by choosing different restriction enzymes. But also here, when the sequencing budget is fixed, choosing for higher marker density should go at the expense of either sample size or average sequencing depth.

To simulate a two-locus model, we considered loci *A* and *B* with alleles *A*_*1*_ *, A*_*2*_ and *B*_*1*_ *, B*_*2*_, and haplotype frequencies of *A*_*1*_ *B*_*1*_ = *x*_*11*_ *, A*_*1*_ *B*_*2*_ = *x*_*12*_ *, A*_*2*_ *B*_*1*_ = *x*_*21*_ *, A*_*2*_ *B*_*2*_ = *x*_*22*_. This gives allele frequencies for allele *A*_*1*_ as *p*_*1*_ = *x*_*11*_ + *x*_*12*_, allele *A*_*2*_ as *p*_*2*_ = *x*_*21*_ + *x*_*22*_, allele *B*_*1*_ as *q*_*1*_ = *x*_*11*_ + *x*_*21*_ and allele *B*_*2*_ as *q*_*2*_ = *x*_*12*_ + *x*_*22*_. LD was expressed here as the correlation (*r*) between genotypes, but in order to simulate haplotypes we needed the basic measure of LD as a covariance (*D*), where the relation between *r* and *D* is (Falconer 1996):

11 $$r = \frac{D}{{\sqrt {\left( {p_{1} q_{1} } \right)(p_{2} q_{2} )} }}$$

Equation (11) allows, for a desired level of *r*, to determine *D*, and to set the haplotype frequencies as:

$$x_{11} = \, p_{1} q_{1} + \, D, \, x_{21} = \, p_{2} q_{1} {-} \, D, \, x_{12} = \, p_{1} q_{2} {-} \, D{\text{ and }}x_{22} = \, p_{2} q_{2} + \, D$$

For the F2 pool genotypes, we sampled four haplotypes from each family and obtained the 4-allele family-pool genotypes for the loci A and B. Phenotypes were simulated as for the one-locus study, but locus A was used as the causal locus to generate phenotypes, while locus B was used as the observed marker locus.

The LD levels in ryegrass appear to be relatively strong for distances <1 Kb, but dropping fast beyond a few Kb (Byrne et al. 2013). This led us to consider scenarios where we doubled the sequencing depth twice (7, 15 and 30 depth), which at total fixed sequenced budget means halving the marker density twice and corresponded to a relatively steep decline in LD. We choose the corresponding LD levels *r* = 0.95, 0.7 and 0.3.

In these simulations, we set sample size to 2,000, used an environmental standard deviation of 4, and allele frequency of 0.1, 0.3 and 0.5. The whole procedure was replicated 1,000 times and the numbers of significant effects were counted. R script for these simulations is provided in supplementary material.

## Results

### Estimation of allele effect in one-locus model

**Fig. 2:**
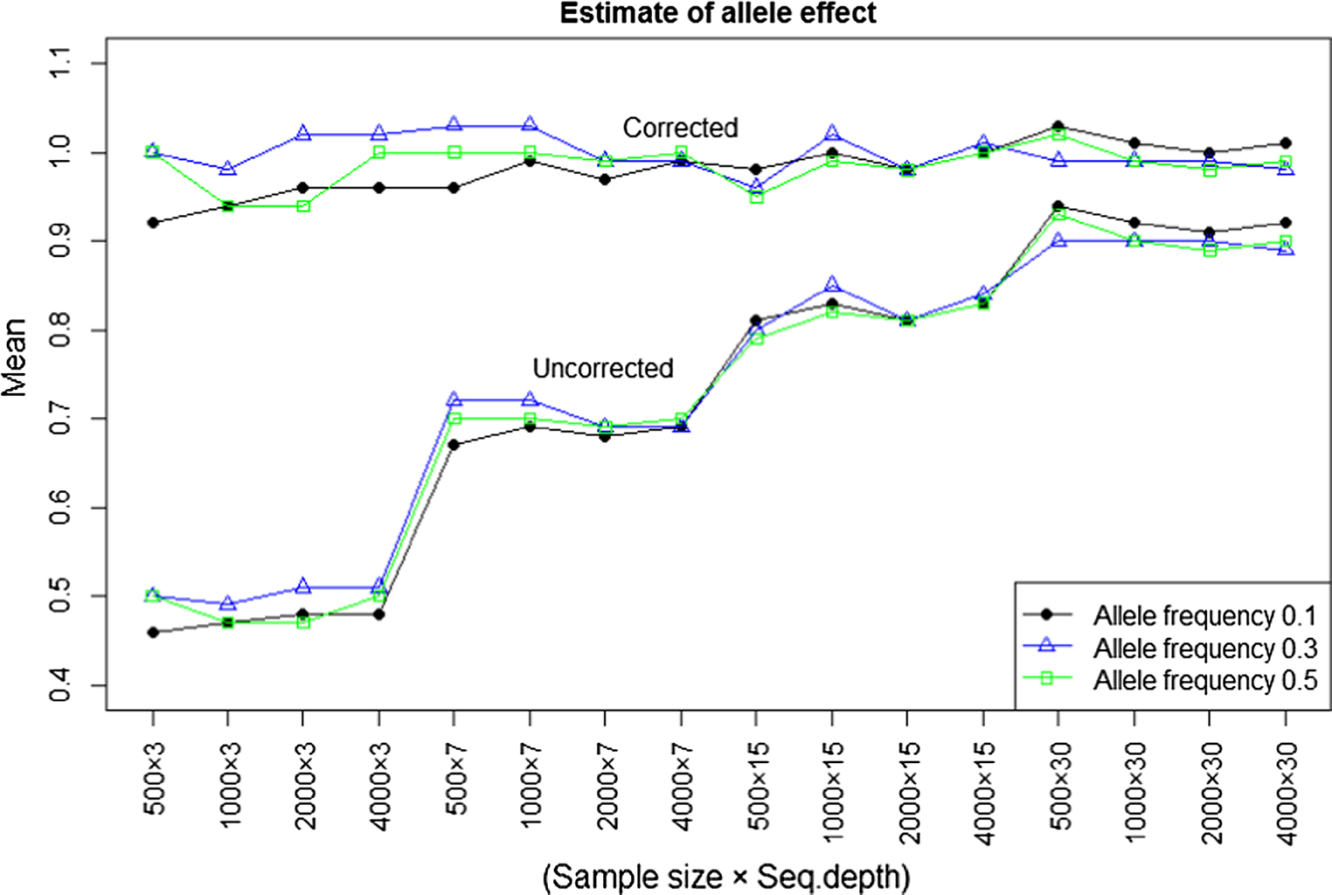
Averages estimated allele effects in a one-locus model. Estimate of allele effect (*uncorrected three lines*) was computed at three different levels of allele frequencies (0.1, 0.3, 0.5), with environmental standard deviation 4. *Corrected* *three lines* are based on applying the derived theoretical expression (10) for bias from using GBS. The true generated allele effect was 1

Figure 2 presents estimated allele effects in a one-locus model from simulated F2-family phenotypes and GBS genotypes, by regressing the pool phenotypes on the pool allele-frequency estimates. The results present allele effect estimates for different sample size, sequencing depth and allele frequency, and every point in the graph is based on 1,000 replicates. The true allele effect in these simulations was 1. Results show a quite severe underestimation of the allele effect at very low sequencing depth (around 0.5 at depth 3), and also at depth 30 some bias remains. Figure 2 also presents corrected estimates by applying the derived formula for the bias from measurement error. As can be seen, the corrected estimates are very close to the true ones with only a small remaining underestimation at very low sequencing depth and low allele frequency. Overall, the underestimation of allele effect depends very little on allele frequency.

We also computed an estimate of the allele effect with environmental standard deviation 10; the results showed that this leads to more underestimation in the estimate of allele effect (Table 1 in supplementary material). Further, we also computed the same for true underlying frequencies in the families, which showed almost the same trend at different levels of sample size and allele frequencies. A full version of the simulation results can be seen in supplementary material Table 1.

### One-locus model power study

**Fig. 3:**
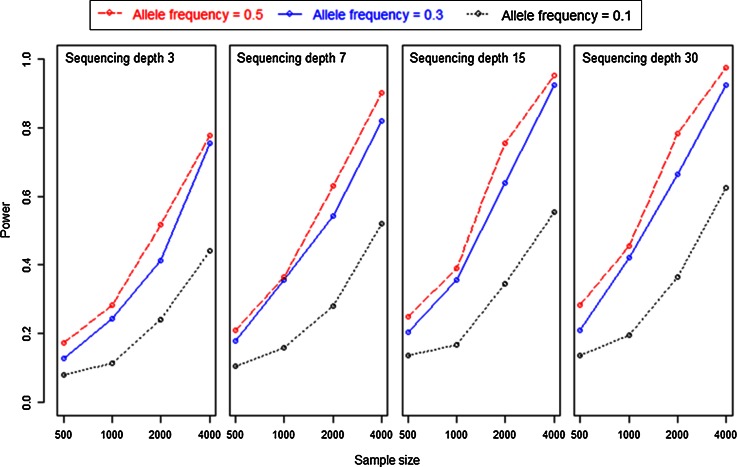
Power to detect a single gene associated with a marker using GBS. The *x*-axis shows sample size and *y*-axis indicates the power (estimate of the probability) from 1,000 replicates. We used four sequencing depths (3, 7, 15, and 30). The *lines*, *red*, *blue* and *black*, show the number of significant results at allele frequency 0.5, 0.3 and 0.1 respectively at environmental standard deviation 4. Here, we used observed frequencies in the families when applying regression of F2 phenotype on F2 pool genotype expression (9)

To study the optimal design for an association study, we performed simulation studies where we varied sample size, sequencing depth and allele frequencies in one locus model. The simulation results for this setup can be seen in Fig. 3.

Figure 3 shows that sample size has a large effect on power, from nearly no power with sample size 500 to around 80–90 % power (for the intermediate allele frequencies) for sample size 4,000. A secondary important factor is allele frequencies showing reduced power at allele frequency 0.1 compared to the other two frequencies. There is not much difference in the power at allele frequencies 0.3 and 0.5. Sequencing depth, finally, has the smallest effect on power: from the left panel at sequencing depth 3 to the right panel at sequencing depth 30, power only increases marginally. The largest differences in power are in the middle range, for instance at sample size 2,000 power increases from about 40 % at sequencing depth 3 to about 60 % at sequencing depth 30.

By adding more environmental variance, i.e., using an environmental standard deviation of 10, we obtained the expected result, .i.e., the power to detect a single gene decreased. Complete results of this simulation study are available in supplementary material Table 2.

**Fig. 4:**
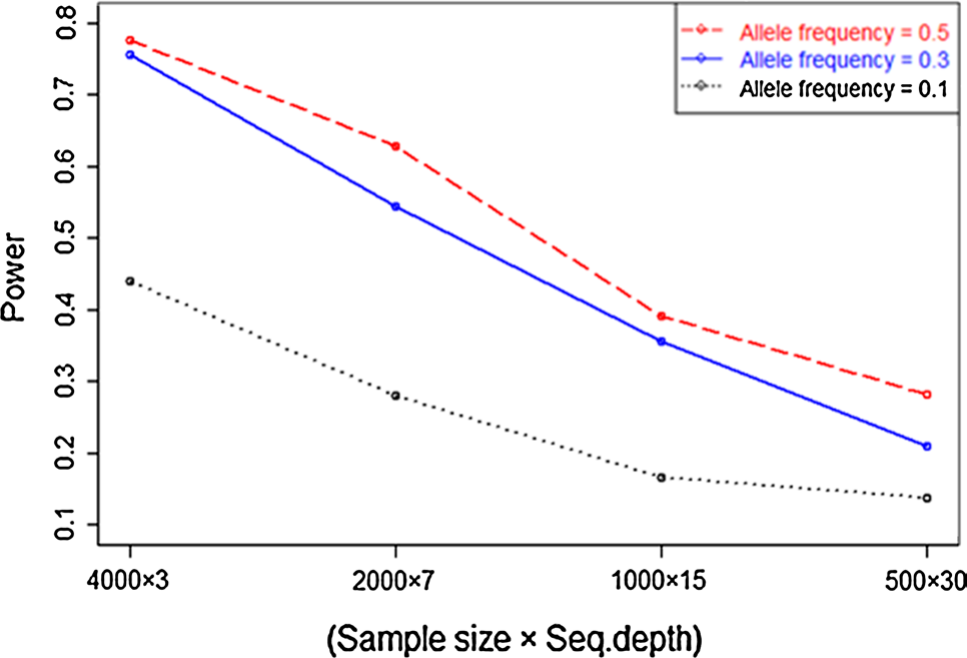
Power to detect a single gene associated with a marker at (almost) equal sequencing efforts in simulation studies. The *x*-axis is the sample size times sequencing depth and *y*-axis is the power (estimate of probability) from 1,000 replicates. *Three lines*, *red*, *blue* and *black*, depict the power at three levels of allele frequencies (0.5, 0.3 and 0.1). (Subset of results presented in Fig. 3)

The results from Fig. 3 are presented again in Fig. 4 by selecting cases with approximately equal sequencing effort, hence approximately equal costs. This optimizes power for a given budget. Results show that power is higher at larger sample size (4,000) and low sequencing depth (3), and starts decreasing by increasing the sequencing depth and reducing sample sizes. There is not much difference in the power at allele frequencies 0.5 and 0.3 while at 0.1, power is comparatively lower.

Overall, this simulation results show that the power to detect a single gene associated with a marker is highest when using the larger sample size at the expense of sequencing depth, similarly in the situation of fixed sequencing capacity it would be more advantageous to use low sequencing depth and maximize the number of samples.

Like many other sequence data sets, GBS datum is also contaminated with some noise. To realize this situation, we also performed simulations to see how the power to detect a single gene associated with a marker varies in the presence of some additional errors. Here, we employ (almost) equal sequencing efforts to obtain power in the presence of three errors (due to binomial sampling, sequencing and all three i.e., binomial, sequencing and genotype calling errors).

**Fig. 5:**
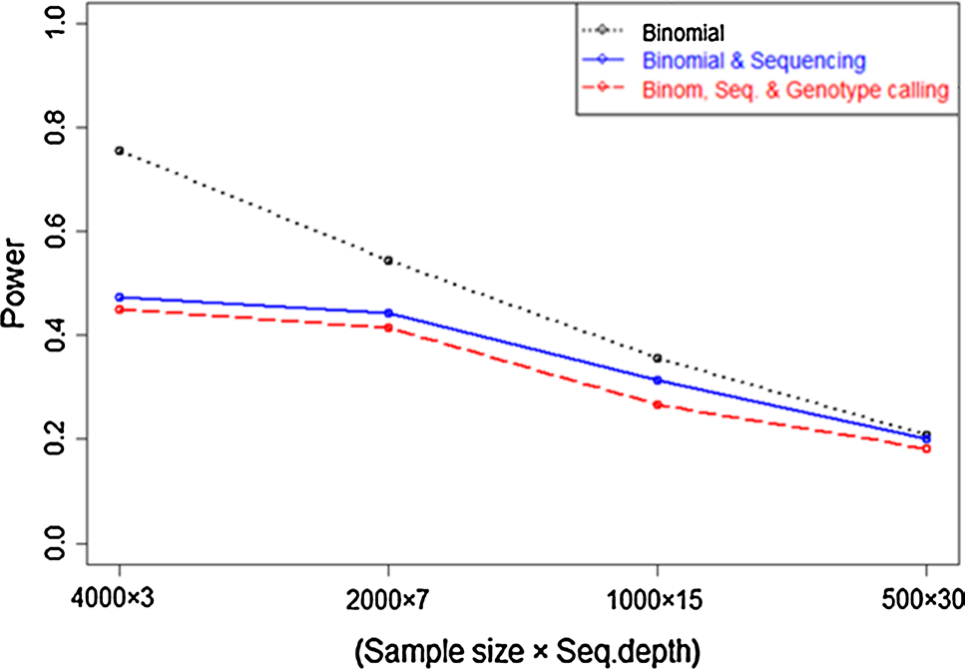
Simulation of the power to detect a single associated with marker in the presence of three errors: binomial sampling, binomial sampling and sequencing error (10 % error rate per read) and all three binomial sampling, sequencing and genotype calling errors at 5 % level of significance. The *x*-axis is the number of families by sequencing depth per family. The unequal sequencing depth was simulated assuming Poisson distribution with mean depth of 3, 7, 15 and 30× against family sizes 4,000, 2,000, 1,000 and 500, respectively. The *black dotted line* corresponds to the power in the presence of only binomial sampling error; the *blue line* is the power if there are two errors, i.e. binomial sampling and sequencing errors; the *red* indicates the power when we incorporate all three errors, i.e. binomial, sequencing and genotype calling errors. The environmental standard deviation was used to be 4

Results (Fig. 5) show that at larger sample size power is 0.76 if there is only noise due to binomial sampling, by adding more noise due to sequencing, the power decreased to 0.47 and even it reduced to 0.45 by incorporating additional noise from genotype calling. Results also indicate that in the situation of fixed sequencing budget, it is more pertinent to increase the number of samples at the expense of sequencing depth.

### Two-locus model power study

To optimize the SNP density given a fixed sequencing budget, we performed simulation studies in a two-locus model, where we consider the case that a marker is linked to a causative mutation. In this power study, we varied SNP density [LD (*r*) stronger to weak] versus sequencing depth (small to large). Like in the one-locus model power study, we used three levels of allele frequencies of 0.1, 0.3 and 0.5, with environmental standard deviations of 2 and 4, and a sample size 2,000. The results of this simulation can be seen in Table 2.

**Table 2:** Power to detect a significant association (number significant from 1,000 replicates) when the measured SNP is not causal and has different levels of LD with a causal locus

Env. SD	Allele freq	Power at LD (*r*) and sequencing depth (*D*)			Power at causal locus
		*r* = 0.95, *D* = 7	*r* = 0.7, *D* = 15	*r* = 0.3, *D* = 30	
2	0.1	0.696	0.534	0.153	0.791
2	0.3	0.973	0.874	0.249	0.984
2	0.5	0.996	0.945	0.346	0.997
4	0.1	0.248	0.197	0.086	0.281
4	0.3	0.533	0.359	0.112	0.544
4	0.5	0.601	0.446	0.155	0.629

The LD levels are chosen to approximately represent steps of halving the SNP density, allowing sequencing at double depth for every step

Table 2 shows that power to obtain a significant association reduces rapidly when LD reduces, and this is not compensated by the higher sequencing depth at lower LD levels. Also here, sequencing depth is the minor factor determining power. Comparison with analysis of the causal locus shows that power is not much reduced when having a linked locus at *r* = 0.95, and is also still reasonable with a linked locus at *r* = 0.7.

### Power as a function of LD and sample size

We also performed simulations to investigate how the power depends on sample size (with almost equal sequencing efforts) at different levels of LD. Results (Fig. 5) indicate that the power to achieve significant association decreased when LD levels reduce, and given allele frequencies are less important to obtain the higher power.

**Fig. 6:**
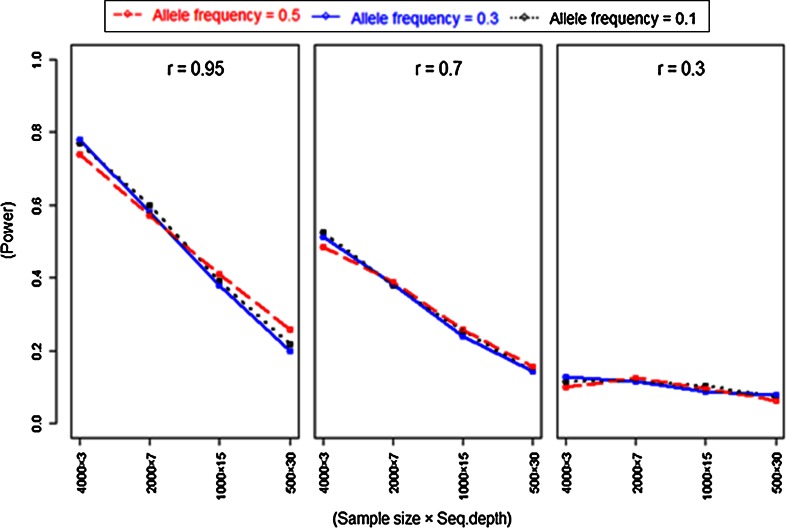
Power as a function of LD and sample size, to obtain significant association when the measured SNP is not causal and has different levels of LD with a causal locus. The LD levels are chosen in such a way that it represent halving the SNP density at each step, allowing (almost) equal sequencing efforts. Three levels (0.5, 0.3 and 0.1) of allele frequencies were used at environmental standard deviation 4 from 1,000 replicates. Complete results (also with environmental standard deviation 2) are supplied in supplementary material (Table 3)

These results revealed that within the constraints of a fixed sequencing budget, higher power can be obtained using higher SNP density, leading to higher LD with causal loci, i.e. by choosing a more frequently cutting restriction enzyme in the GBS technique (Fig. 6).

## Discussion

We have developed theory and models to perform association studies in F2-family pools where the “pool genotype” is obtained as an allele-frequency estimate from GBS. The GBS technology is getting more and more interesting due to dropping prices for sequencing and lends itself flexibly to genotype either individuals or pools. Use of GBS on pools is especially attractive, because GBS produces allele-frequency estimates (Byrne et al. 2013), which is the most logical approach to process pool genotyping data from outbreeding plants. Other studies proposing the use of GBS have considered to explicitly call genotypes, but relatively large sequencing depth is required to minimize errors for genotype calling. Chenuil (2012) computed that accurate calling of heterozygotes in diploid species needs a minimum sequencing depth of around 10×. The situation gets much worse for tetraploids, which would also apply to the F2-family pools in our study: accurate calling of tetraploid genotypes requires sequencing depths of 60–80× (Uitdewilligen et al. 2013). At lower sequencing depths, the called genotypes would show considerable measurement error. Although it might be possible also to deal with that error on a genotype level, our approach to use allele frequencies and to deal with the error on the frequency estimates is much more logical and straightforward. Our approach to analyze F2-family pools could directly be applied for association studies in polyploids. We have argued that the genotype of an F2 pool (here from diploid parents) is conceptually the same as a tetraploid genotype (Björn et al. 2010). The proposed association model using GBS data is a regular linear regression model, allowing to add environmental covariates, gene–gene and gene–environment interactions, and to fit the model in standard software packages. Also, multi-locus models may be considered, and we anticipate that it will be useful to include our bias terms as ‘weights’ in multi-locus models to correct for differences between SNPs in sequencing depth. Other species with similar breeding schemes may use F1 full-sib families or F1 half-sib families. Such other types of family pools could be accommodated in our association model framework.

In our derivation of expected pool genotypes, we have relied on knowledge of the genetic origin, i.e., we assumed that pools originate from the crossing of F1 parents. As a background for the developed model we have considered a perennial ryegrass breeding program. The F1 propagation in perennial ryegrass breeding is done in open fields, with the theoretical possibility of pollination from other F1 plots. Such cross-pollination would reduce the genetic variance between pools (making F2 pools more alike), and could in principle be assessed and included in statistical models when also the parents are genotyped. However, typically pollen barriers of other tall crops are placed between the F1 plots to minimize pollination from other fields. Also, other factors may change or distort the allele frequencies in the pools, for instance genetic drift, selection, and linkage to self-incompatibility loci. We argue that a strong point in the approach to sequence pools is that any such distortion is directly measured and will be taken into account. An approach to call genotypes would be less robust against such distortions, because it would force the frequencies to quarters, even when that may not be correct.

Sequencing is not perfect, but current Next-Generation Sequencing technology allows to accurately compute an individual base-call error rate (Phred scores; Illumina 2011). A common QC would only accept base reads with a probability for an incorrect base call below 1/1,000 (Phred score > 30). We assessed the effect of sequencing error at a much higher level, which indeed showed a reduction in power due to sequencing errors. Also the calling of genotypes, which forces the (semi-)continuous frequencies to quarters, reduces power slightly.

In the theory derived here, we have not considered relationships between parents, inbreeding between the F1’s, dominance, and drift in the F1 replication. As we have noted, relationships between parents can be inserted by modifying Eq. (4) to include a covariance term between the parental genotypes. The intercrossing between F1 full sibs will cause inbreeding in the F2’s, but this inbreeding could be ignored because the analysis is based on F2 mean genotypes and phenotypes and on additive models. The inbreeding in F2 will increase variance between the individuals in the F2 family, but under an additive model this does not change the family mean phenotype or genotype. Extending the model to include dominance, however, would need to account for this inbreeding in the F2, and would require that the level of self- and cross-pollination is known. Perennial ryegrass is mostly cross-pollinating and may also show some self-pollination, depending on alleles at self-incompatibility loci (Huff 1997; Thorogood et al. 2002). This makes it non-trivial to correctly determine this inbreeding and derive proper estimates for dominance effects in perennial ryegrass F2-family pools. In principle, there is also genetic drift in the F1 replication, but the F1 × F1 replication is based on at least 100 plants so that drift should be small.

In our theory we have derived an expression for the measurement error on allele frequencies obtained from GBS data. This measurement error leads to an underestimation of the allele effect when using GBS data. The theoretical derivation of the measurement error showed that the underestimation should not depend on allele frequency. The simulation studies showed a very small deviation from the theory for low sample size and low allele frequency, but overall our theoretical expression is adequate to describe and correct for the underestimation in the allele effect estimates. The correction for measurement error can be used to provide corrected, thus comparable, allele effect estimates across SNPs, across studies, or for prediction models. In our power studies, we have not explicitly considered the underestimation of allele frequency from measurement error in the pool genotypes as this is not relevant to assess significance and power. Measurement error is well studied in several areas of especially social research and is known to create potentially complicated biases when models become more complex (Bekker 1986; Chesher 1991). In our study, we were able to derive an expression for this measurement error from knowledge on the underlying genetics of F2-family pools. When the genetic background of pools or varieties become less clear, for instance when multiple parents contribute to a variety in unknown proportions, additional approaches may be used to derive or assess the measurement error (Fuller 1987; Divers et al. 2007; Padilla et al. 2009).

The power studies show that sequencing depth is the least critical parameter in achieving large power. Therefore, it is advantageous to increase sample size and/or SNP density at the expense of a lower sequencing depth. We verified that in theory sequencing depth may even be as low as 2 reads/sample, if this would be compensated by larger sample size. Some other studies (Pasaniuc et al. 2012) also suggested the advantages of even lower sequencing depth. However, due to variation in the sequencing depth over the genome, this would lead to many missing genotypes. One of the main issues of using GBS is missing data (Beissinger et al. 2013); therefore, we recommend using a sequencing depth which is just sufficient to minimize missing data. Assuming a Poisson distribution for the number of reads sequenced per sample, the probability to have no reads is 0.7 % at average depth 5, 0.2 % at average depth 6, 0.1 % at average depth 7, etc. In practice the missing rates are higher due to additional variation, but first experiences with GBS data on pools of ryegrass varieties (Byrne et al. 2013) indicate that missing rates are manageable at average depth between 5× and 10×.

## Electronic supplementary material

Below is the link to the electronic supplementary material.

Supplementary material 1 (DOCX 42 kb)
