# Supplementary material for: Association studies using family pools of outcrossing crops based on allele-frequency estimates from DNA sequencing
Source: Theor Appl Genet. 2014 Mar 26;127(6):1331–41. doi: 10.1007/s00122-014-2300-4 (PMC4035547; doi:10.1007/s00122-014-2300-4)
Supplement: Supplementary file 1 — Supplementary material 1 (DOCX 42 kb) [file 122_2014_2300_MOESM1_ESM.docx]

**Supplementary material: Tables and R Scripts**

**Association studies using family pools of outcrossing crops based on allele frequency estimates from DNA sequencing**

Bilal H. Ashraf^1*^, Just Jensen^1^, Torben Asp^2^, Luc L. Janss^1^

1- Centre for Quantitative Genetics and Genomics, Dept. of Molecular Biology and Genetics, Aarhus University, Blichers Alle 20, Post Box 50, DK-8830 Tjele, Denmark. 2- Dept. of Molecular Biology and Genetics, Aarhus University, Forsogsvej 1 DK-4200 Slagelse, Denmark

* Author to whom correspondence should be addressed; E-mail: bilalh.ashraf@agrsci.dk; Tel.: +45 87157972; Fax: +45 87154994.

Journal name: Theoretical and Applied Genetics (TAG)

1. **Table 3** Mean and standard deviation of the estimated allele effect in one locus model

| **Sample size – sequencing depth** | **Model** | **Mean/Standard deviation** | | | | | |
| --- | --- | --- | --- | --- | --- | --- | --- |
|  |  | **Environmental SD 4** | | | **Environmental SD 10** | | |
|  |  | **Allele frequency** | | | **Allele frequency** | | |
|  |  | **0.1** | **0.3** | **0.5** | **0.1** | **0.3** | **0.5** |
| 500 - 3 | y ~ o | 0.46/0.88 | 0.50/0.55 | 0.50/0.49 | 0.41/2.22 | 0.42/1.38 | 0.48/1.22 |
|  | y ~ t | 1.04/1.26 | 0.97/0.78 | 0.95/0.68 | 0.89/2.70 | 1.01/2.09 | 1.00/1.82 |
| 1000 - 3 | y ~ o | 0.47/0.60 | 0.49/0.38 | 0.47/0.37 | 0.46/1.47 | 0.47/1.01 | 0.45/0.91 |
|  | y ~ t | 1.01/0.88 | 1.02/0.54 | 0.99/0.51 | 1.01/2.13 | 0.98/1.37 | 1.01/1.18 |
| 2000 - 3 | y ~ o | 0.48/0.42 | 0.51/0.28 | 0.47/0.25 | 0.46/1.04 | 0.50/0.68 | 0.49/0.63 |
|  | y ~ t | 0.98/0.58 | 0.98/0.40 | 1.01/0.35 | 0.98/1.46 | 1.00/0.96 | 1.01/0.92 |
| 4000 - 3 | y ~ o | 0.48/0.31 | 0.51/0.19 | 0.50/0.17 | 0.53/0.76 | 0.46/0.48 | 0.49/0.44 |
|  | y ~ t | 1.01/0.42 | 0.99/0.27 | 1.00/0.26 | 1.07/1.03 | 1.00/0.66 | 0.97/0.64 |
| 500 – 7 | y ~ o | 0.67/1.02 | 0.72/0.67 | 0.70/0.60 | 0.65/2.32 | 0.68/1.54 | 0.62/1.55 |
|  | y ~ t | 0.98/1.17 | 0.99/0.77 | 0.98/0.71 | 0.87/3.14 | 0.91/2.00 | 0.90/1.79 |
| 1000 – 7 | y ~ o | 0.69/0.67 | 0.72/0.45 | 0.70/0.42 | 0.64/1.88 | 0.75/1.18 | 0.71/1.06 |
|  | y ~ t | 1.02/0.81 | 0.99/0.57 | 1.00/0.52 | 0.99/2.06 | 1.02/1.40 | 0.96/1.25 |
| 2000 – 7 | y ~ o | 0.68/0.50 | 0.69/0.33 | 0.69/0.31 | 0.70/1.21 | 0.67/0.81 | 0.69/0.71 |
|  | y ~ t | 1.05/0.61 | 0.97/0.38 | 1.01/0.37 | 0.95/1.50 | 1.01/0.94 | 0.96/0.89 |
| 4000 - 7 | y ~ o | 0.69/0.36 | 0.69/0.23 | 0.70/0.20 | 0.68/0.87 | 0.71/0.58 | 0.70/0.55 |
|  | y ~ t | 0.98/0.40 | 1.02/0.27 | 0.99/0.26 | 1.00/1.01 | 1.01/0.69 | 0.99/0.64 |
| 500 – 15 | y ~ o | 0.81/1.05 | 0.80/0.76 | 0.79/0.68 | 0.81/2.71 | 0.83/1.83 | 0.75/1.65 |
|  | y ~ t | 1.00/1.26 | 1.00/0.79 | 0.97/0.66 | 0.85/2.93 | 0.94/1.96 | 0.86/1.81 |
| 1000 – 15 | y ~ o | 0.83/0.75 | 0.85/0.49 | 0.82/0.47 | 0.89/1.95 | 0.80/1.31 | 0.85/1.17 |
|  | y ~ t | 0.97/0.82 | 0.98/0.54 | 1.02/0.49 | 0.95/2.32 | 1.05/1.36 | 1.01/1.25 |
| 2000 - 15 | y ~ o | 0.81/0.56 | 0.81/0.35 | 0.81/0.31 | 0.77/1.39 | 0.83/0.90 | 0.82/0.77 |
|  | y ~ t | 1.01/0.58 | 1.00/0.40 | 1.02/0.36 | 1.07/1.41 | 0.96/0.95 | 1.02/0.90 |
| 4000 – 15 | y ~ o | 0.83/0.36 | 0.84/0.25 | 0.83/0.23 | 0.81/0.95 | 0.88/0.63 | 0.82/0.55 |
|  | y ~ t | 0.98/0.41 | 1.02/0.28 | 0.99/0.25 | 1.00/1.04 | 0.98/0.69 | 1.01/0.64 |
| 500 – 30 | y ~ o | 0.94/1.06 | 0.90/0.75 | 0.93/0.69 | 0.79/2.76 | 0.93/1.80 | 0.94/1.59 |
|  | y ~ t | 0.95/1.13 | 0.98/0.79 | 1.02/0.73 | 1.07/2.97 | 0.94/1.89 | 1.04/1.73 |
| 1000 – 30 | y ~ o | 0.92/0.79 | 0.90/0.53 | 0.90/0.50 | 0.92/1.91 | 0.86/1.28 | 0.89/1.23 |
|  | y ~ t | 1.02/0.81 | 0.99/0.53 | 0.98/0.48 | 1.00/2.09 | 0.97/1.30 | 0.96/1.35 |
| 2000 – 30 | y ~ o | 0.91/0.52 | 0.90/0.37 | 0.89/0.34 | 0.94/1.41 | 0.93/0.93 | 0.91/0.84 |
|  | y ~ t | 1.03/0.59 | 1.00/0.40 | 0.98/0.36 | 0.98/1.48 | 0.99/0.92 | 1.03/0.94 |
| 4000 - 30 | y ~ o | 0.92/0.39 | 0.89/0.25 | 0.90/0.25 | 0.92/0.98 | 0.90/0.63 | 0.91/0.61 |
|  | y ~ t | 1.00/0.42 | 1.01/0.27 | 0.99/0.25 | 0.97/1.03 | 1.02/0.70 | 0.99/0.65 |

Note. “y ~ o”; model on observed frequencies and “y ~ t”; model on true frequencies

1. **R script for estimated allele effect (for table 3)**

F2pool<- rbinom(1000, size=4,p=0.3) **# generation of F2 pool genotypes**

F2poolGenotype<- F2pool/4

SIM <- function(F2poolGenotype){ **# simulation and analysis**

ObservedFrequencies <- (rbinom(1000,10,F2poolGenotype))/10

Env <- rnorm(2000,0,4) **# environmental standard deviation**

Phenotype <- F2poolGenotype + Env **# regression of F2 phenotype of F2 pool genotype**

s <- summary(lm(Phenotype ~ ObservedFrequencies))$coefficients["ObservedFrequencies","Estimate"]

return(s) } **# obtain estimate of allele effect**

Estimateofallele <- unlist(replicate(1000, SIM(F2poolGenotype))) **# repeated 1000 times**

mean(Estimateofallele) **# mean and standard deviation**

sd(Estimateofallele)

Note: In this simulation we varied sample size, allele frequency, sequencing depth and environmental standard deviation. We also computed the same for true underlying frequencies in F2 family pool by modeling F2 phenotype on F2 pool genotypes without environmental standard deviation. By using true underlying frequencies, results are available in above table 3, but here our main emphasis would be on results obtained by using observed frequencies.

1. **Table 4** Power to detect a single gene associated with a marker using GBS in simulation studies for one locus model at different levels of sample size, sequencing reads depth, environmental standard deviation and allele frequencies

| **Sample size – sequencing depth** | **Model** | **Significance** | | | | | |
| --- | --- | --- | --- | --- | --- | --- | --- |
|  |  | **Environmental SD 4** | | | **Environmental SD 10** | | |
|  |  | **Given Allele frequency** | | | **Given Allele frequency** | | |
|  |  | **0.1** | **0.3** | **0.5** | **0.1** | **0.3** | **0.5** |
| 500 - 3 | y ~ o | 0.08 | 0.128 | 0.172 | 0.059 | 0.071 | 0.064 |
|  | y ~ t | 0.139 | 0.283 | 0.308 | 0.063 | 0.087 | 0.099 |
| 1000 - 3 | y ~ o | 0.114 | 0.243 | 0.283 | 0.068 | 0.071 | 0.096 |
|  | y ~ t | 0.177 | 0.454 | 0.475 | 0.074 | 0.122 | 0.125 |
| 2000 - 3 | y ~ o | 0.239 | 0.412 | 0.517 | 0.077 | 0.096 | 0.140 |
|  | y ~ t | 0.383 | 0.723 | 0.807 | 0.102 | 0.167 | 0.190 |
| 4000 - 3 | y ~ o | 0.440 | 0.756 | 0.777 | 0.097 | 0.166 | 0.191 |
|  | y ~ t | 0.670 | 0.954 | 0.982 | 0.177 | 0.290 | 0.360 |
| 500 - 7 | y ~ o | 0.106 | 0.178 | 0.209 | 0.057 | 0.082 | 0.081 |
|  | y ~ t | 0.143 | 0.244 | 0.299 | 0.061 | 0.077 | 0.091 |
| 1000 - 7 | y ~ o | 0.158 | 0.356 | 0.364 | 0.065 | 0.077 | 0.105 |
|  | y ~ t | 0.218 | 0.438 | 0.499 | 0.071 | 0.098 | 0.120 |
| 2000 - 7 | y ~ o | 0.281 | 0.544 | 0.629 | 0.072 | 0.130 | 0.158 |
|  | y ~ t | 0.387 | 0.691 | 0.805 | 0.102 | 0.153 | 0.199 |
| 4000 - 7 | y ~ o | 0.521 | 0.820 | 0.901 | 0.107 | 0.239 | 0.263 |
|  | y ~ t | 0.712 | 0.971 | 0.973 | 0.164 | 0.273 | 0.324 |
| 500 - 15 | y ~ o | 0.135 | 0.203 | 0.250 | 0.059 | 0.074 | 0.086 |
|  | y ~ t | 0.134 | 0.261 | 0.259 | 0.058 | 0.080 | 0.077 |
| 1000 - 15 | y ~ o | 0.166 | 0.356 | 0.391 | 0.065 | 0.109 | 0.098 |
|  | y ~ t | 0.214 | 0.427 | 0.515 | 0.085 | 0.108 | 0.100 |
| 2000 - 15 | y ~ o | 0.345 | 0.639 | 0.754 | 0.110 | 0.135 | 0.175 |
|  | y ~ t | 0.393 | 0.715 | 0.821 | 0.110 | 0.174 | 0.199 |
| 4000 - 15 | y ~ o | 0.555 | 0.923 | 0.952 | 0.141 | 0.252 | 0.295 |
|  | y ~ t | 0.695 | 0.950 | 0.971 | 0.169 | 0.290 | 0.371 |
| 500 - 30 | y ~ o | 0.137 | 0.209 | 0.282 | 0.049 | 0.078 | 0.089 |
|  | y ~ t | 0.131 | 0.252 | 0.298 | 0.057 | 0.083 | 0.078 |
| 1000 - 30 | y ~ o | 0.195 | 0.421 | 0.454 | 0.085 | 0.101 | 0.133 |
|  | y ~ t | 0.226 | 0.420 | 0.548 | 0.074 | 0.113 | 0.106 |
| 2000 - 30 | y ~ o | 0.365 | 0.664 | 0.784 | 0.084 | 0.186 | 0.168 |
|  | y ~ t | 0.399 | 0.721 | 0.791 | 0.097 | 0.188 | 0.226 |
| 4000 - 30 | y ~ o | 0.624 | 0.925 | 0.975 | 0.167 | 0.316 | 0.336 |
|  | y ~ t | 0.677 | 0.946 | 0.979 | 0.164 | 0.290 | 0.374 |

1. **Table 6** Power as a function of LD and sample size, to detect a significant association (from 1000 replicates) when the measured SNP is not causal and has different levels of LD with a causal locus.

| **(Sample size × Seq.depth)** | **Env.SD** | **Power at LD (r) and sample size (with equal sequencing efforts)** | | | | | | | | |
| --- | --- | --- | --- | --- | --- | --- | --- | --- | --- | --- |
|  |  | **0.95** | | | **0.7** | | | **0.3** | | |
|  |  | **Allele frequency** | | | **Allele frequency** | | | **Allele frequency** | | |
|  |  | **0.1** | **0.3** | **0.5** | **0.1** | **0.3** | **0.5** | **0.1** | **0.3** | **0.5** |
| (500 × 30) | 4 | 0.217 | 0.198 | 0.259 | 0.144 | 0.142 | 0.154 | 0.070 | 0.078 | 0.062 |
|  | 2 | 0.686 | 0.699 | 0.702 | 0.472 | 0.487 | 0.435 | 0.144 | 0.141 | 0.098 |
|  |  |  |  |  |  |  |  |  |  |  |
| (1000 × 15) | 4 | 0.392 | 0.378 | 0.409 | 0.256 | 0.238 | 0.258 | 0.103 | 0.086 | 0.097 |
|  | 2 | 0.911 | 0.919 | 0.927 | 0.694 | 0.683 | 0.683 | 0.197 | 0.153 | 0.140 |
|  |  |  |  |  |  |  |  |  |  |  |
| (2000 × 7) | 4 | 0.599 | 0.581 | 0.571 | 0.378 | 0.382 | 0.389 | 0.114 | 0.115 | 0.124 |
|  | 2 | 0.995 | 0.988 | 0.994 | 0.918 | 0.925 | 0.876 | 0.275 | 0.243 | 0.246 |
|  |  |  |  |  |  |  |  |  |  |  |
| (4000 × 3) | 4 | 0.769 | 0.778 | 0.739 | 0.525 | 0.513 | 0.483 | 0.115 | 0.127 | 0.100 |
|  | 2 | 1 | 1 | 1 | 0.976 | 0.981 | 0.975 | 0.402 | 0.372 | 0.298 |

1. **R script for one locus model power study (for table 4)**

Here we are varied sample size, allele frequency, sequencing depth and environmental standard deviation level.

F2pool<- rbinom(1000, size=4,p=0.3) **# generation of F2 pool genotypes**

F2poolGenotype<- F2pool/4

SIM <- function(F2poolGenotype){ **# simulation and analysis**

ObservedFrequencies <- (rbinom(1000,10,F2poolGenotype))/10

Env <- rnorm(2000,0,4) **# environmental standard deviation**

Phenotype <- F2poolGenotype + Env **# regression of F2 phenotype on F2 pool genotype**

s <- summary(lm(Phenotype ~ ObservedFrequencies))[[4]][[8]]

return(s) } **# obtained p-values**

Replicate <- unlist(replicate(1000, SIM(F2poolGenotype))) **# repeated 1000 times**

Replicate[Replicate <=0.05] **# no. of significants**

1. **R script for two locus model power study (for table 5 in Paper )**

Here we varied SNP density against sequencing depth at sample size 2000.

p1<- 0.3

q1<- 0.7

p2<- 0.3 **# corresponding allele frequencies of two locus**

q2<- 0.7

r<- 0.95

D<- r*sqrt(p1*q1*p2*q2) **# level of linkage disequilibrium**

x11 <- p1*q1 + D

x21 <- p2*q1 - D **# haplotype frequencies**

x12 <- p1*q2 - D

x22 <- p2*q2 + D

F2poolGenotype<- rmultinom(1000, size=4, prob=c(x11, x21, x12, x22))/4  **#Generation of F2 pool genotypes**

A2<- c(0,1,0,1)

A2allele<- matrix(A2, 1, 4, byrow=T)

Agenotype<- A2allele%*%F2poolGenotype

B2<- c(0,0,1,1)

B2allele<- matrix(B2, 1, 4, byrow=T)

Bgenotype<- B2allele%*%F2poolGenotype

cor.test(Agenotype,Bgenotype) **#correlation between two genotypes**

SIM <- function(Agenotype,Bgenotype){ **#Simulation and Analysis**

ObservedFrequencies <- rbinom(1000,30,Agenotype)/30

Bgenotype <- as.vector(Bgenotype)

Env <- rnorm(1000,0,4)

Phenotype <- Bgenotype + Env **#Regression of F2 phenotype**

s <- summary(lm(Phenotype ~ as.vector(ObservedFrequencies)))[[4]][[8]] **# obtained P-values**

return(s)}

Replicate <- unlist(replicate(1000, SIM(Agenotype,Bgenotype))) **# Repeated 1000 time**

Replicate[Replicate <=0.05]
